# Spatial and environmental correlates of species richness and turnover patterns in European cryptocephaline and chrysomeline beetles

**DOI:** 10.3897/zookeys.597.6792

**Published:** 2016-06-09

**Authors:** Andrea Freijeiro, Andrés Baselga

**Affiliations:** 1 Departamento de Zoología, Facultad de Biología, Universidad de Santiago de Compostela, c/ Lope Gómez de Marzoa s/n, 15782 Santiago de Compostela, Spain

**Keywords:** Beta diversity, biogeography, Chrysomelidae, Chrysomelinae, Cryptocephalinae, species richness

## Abstract

Despite some general concordant patterns (i.e. the latitudinal richness gradient), species richness and composition of different European beetle taxa varies in different ways according to their dispersal and ecological traits. Here, the patterns of variation in species richness, composition and spatial turnover are analysed in European cryptocephaline and chrysomeline leaf beetles, assessing their environmental and spatial correlates. The underlying rationale to use environmental and spatial variables of diversity patterns is to assess the relative support for niche- and dispersal-driven hypotheses. Our results show that despite a broad congruence in the factors correlated with cryptocephaline and chrysomeline richness, environmental variables (particularly temperature) were more relevant in cryptocephalines, whereas spatial variables were more relevant in chrysomelines (that showed a significant longitudinal gradient besides the latitudinal one), in line with the higher proportion of flightless species within chrysomelines. The variation in species composition was also related to environmental and spatial factors, but this pattern was better predicted by spatial variables in both groups, suggesting that species composition is more linked to dispersal and historical contingencies than species richness, which would be more controlled by environmental limitations. Among historical factors, Pleistocene glaciations appear as the most plausible explanation for the steeper decay in assemblage similarity with spatial distance, both in cryptocephalines and chrysomelines.

## Introduction

The assessment of large-scale biogeographic patterns has proven a fruitful research discipline for understanding the ecological, evolutionary and historical processes that have determined the current distribution of biological diversity (Brown and Maurer 1989; Farrell 1998; Gaston and Blackburn 2000). In recent years, several contributions have considerably advanced in our search for a general explanation about the factors driving species distributions and clade diversification, using both empirical (Hawkins et al. 2003; Svenning and Skov 2007a; Wiens 2007) and theoretical approaches (Hubbell 2001; McGill 2010). However, probably because of the complexity of the problem and the difficulty to perform controlled experiments due to the large temporal and spatial scale of the processes potentially involved, a general comprehensive theoretical framework to explain biological diversity and its distribution on Earth is still missing. From the empirical point of view, one important shortfall is the under-representation of invertebrates in biogeographical studies, caused by the difficulty to sample and identify invertebrate species, compared to vertebrates or plants. This has led to a marked scarcity of invertebrate distribution data that prevented biogeographers to assess invertebrate biodiversity patterns. As a result, most biogeographical and macroecological theories have been tested using vertebrate and plant data, a circumstance that does not fit very well with the fact that invertebrates are the largest fraction of local, regional or global biodiversity (Erwin 1982; Gaston 1991; Odegaard 2000; Storck 1997). Therefore, a potential avenue for progressing towards a full understanding of biodiversity patterns would be to gather invertebrate distribution data and assess large-scale biodiversity patterns.

Recent examples of this approach have proven particularly successful. A few invertebrate taxa (e.g., diurnal Lepidoptera) have been particularly well sampled, so diversity patterns are relatively well known (Hawkins 2010; Hawkins and Porter 2003). In other cases, the acquisition of reliable distributional data requires considerable sampling effort (e.g., Baselga et al. 2013; Papadopoulou et al. 2011), but an alternative solution to go around the impediment caused by the lack of accurate distributional data is to use country level inventories. Despite its limitations derived from the coarse spatial resolution and the unequal country area (Keil and Hawkins 2009), this kind of data has proven robust enough to unveil the major correlates of species richness and turnover patterns, as shown for water beetles (Ribera et al. 2003), longhorn beetles (Baselga 2008), ground beetles (Schuldt and Assmann 2009; Schuldt et al. 2009), springtails (Ulrich and Fiera 2009), Odonata and Lepidoptera (Keil and Hawkins 2009), and darkling beetles (Fattorini and Baselga 2012). Regarding beetles, the important contributions of databases as Fauna Europaea (Fauna Europaea version 2.6, available online at http://www.faunaeur.org) and catalogues as the Catalogue of Palaearctic Coleoptera (Löbl and Smetana 2003; 2004; 2006; 2010) have been crucial for making possible the aforementioned analyses, including the comparative assessment of biodiversity patterns across multiple beetle taxa (Baselga et al. 2012b; Gómez-Rodríguez et al. 2015).

The studies cited above have shown that different beetle taxa have different diversity patterns across continental Europe. Regarding species richness, the steepness of the negative latitudinal richness gradient has been shown to be related to the dispersal capacity of taxa (Baselga et al. 2012b), suggesting a causal link between the loss of species richness to the North and the incomplete re-colonization of northern regions after Pleistocene glaciations (Baselga et al. 2012a; Svenning et al. 2008; Svenning and Skov 2007a). Regarding the variation in species composition (i.e. beta diversity), available evidence suggests that both dispersal capacity and niche traits are responsible for differences in beta diversity patterns among beetle taxa (Gómez-Rodríguez et al. 2015). Within this context, the aim of this study is to assess the biodiversity patterns across continental Europe in two major clades of leaf beetles: Cryptocephalinae (excluding clytrines) and Chrysomelinae. To this end, the variation in species richness, species composition and species spatial turnover has been assessed in both leaf beetle groups, and their relationship with two sets of environmental and spatial variables. The rationale behind is to confront the explanatory capacity of current environmental factors that would reflect the effect of niche-based processes (Hawkins et al. 2003) with purely spatial variables that could reflect alternative dispersal-based processes, as historical colonization events or neutral dynamics (Lobo et al. 2001; Svenning and Skov 2007a).

## Methods

### Data

The study area included continental Europe. Thirty-six inventories of Cryptocephalinae and Chrysomelinae were obtained from Löbl and Smetana (2010). Delimitations of both subfamilies also follow Löbl and Smetana (2010), so, for example, clytrines were not included within Cryptocephalinae. Most of the 36 inventories refer to European countries, but European Russia was divided in three separate territories (northern, central and southern) due to its extremely large area, while Bosnia and Croatia were included in a single checklist. Finally, only the European portion of Turkey was considered here. For simplicity, all these territorial units are hereafter referred to as “countries.” Islands were excluded from this study to avoid insularity effects, which could confound general continental patterns of diversity. In total, 257 cryptocephaline and 328 chrysomeline species (or subspecies) were considered in this study.

Three sets of variables were obtained for each country: (i) area; (ii) spatial position: mean, minimum and maximum longitude (Long, Long_min_, Long_max_), mean, minimum and maximum latitude (Lat, Lat_min_, Lat_max_) and longitudinal and latitudinal range (Long_ran_ and Lat_ran_); and (iii) environmental factors: mean altitude (Alt); altitudinal range (Alt_ran_); annual mean temperature (T_ann_); spatial range of T_ann_ (T_ran_); maximum temperature of the warmest month (T_max_); minimum temperature of the coldest month (T_min_); annual precipitation (P_ann_); spatial range of P_ann_ (P_ran_); precipitation of driest quarter (P_dri_); and spatial range of P_dri_ (P_drn_). Topographic and climatic variables were obtained from WorldClim 1.4 layers (Hijmans et al. 2005). Thereafter, mean or range values for each country were extracted from a European GIS database (0.08 degrees resolution) using IDRISI (Clark Labs 2000), together with their respective areas (km^2^) and geographical coordinates (lat/long).

### Analytical methods

The relationship between diversity attributes (species richness, species composition and spatial turnover) and the aforementioned variables was independently assessed for cryptocephalines and chrysomelines:

*1. Variation in species richness*. Multiple relationships between species richness and the explanatory variables were analysed using regression modelling (Legendre and Legendre 1998) performed with Statistica 7.0 (StatSoft 2004). Linear, quadratic and cubic functions of the variables were independently regressed against each response variable to determine significant relationships. Significant terms for each set (i.e. area [A], environment [E], and spatial variables [S]) were selected by means of a backward stepwise procedure. Finally, to partition the variation in species composition among A, E and S sets of variables, models including all the possible combinations of sets were performed (i.e. A+E, A+S, E+S and A+E+S). This allowed to quantify the relative importance of the unique contributions of area (A), environment (E) and spatial variables (S), and their respective shared variances (Legendre and Legendre 1998). Such an approach allows non-independent explanatory variables to be dealt with, as it is explicitly designed to identify the portions of variation that are jointly accounted for by different sets of variables and those portions that are independently accounted for (Borcard et al. 1992). Area is included as a covariable in order to control for the effect of differences in area among sampling units.

*2. Variation in species composition*. The variation in species composition among countries was analysed with the Simpson’s index of dissimilarity (β_sim_) (Lennon et al. 2001; Simpson 1943). This index quantifies the spatial turnover component of beta diversity, i.e. the dissimilarity caused by the substitution of some species by others, removing the effect of richness differences on beta diversity (Baselga 2010; 2012). A pair-wise dissimilarity matrix based on β_sim_ was computed using command *beta.pair* in R package *betapart* (Baselga and Orme 2012). The patterns of variation in species composition were visually inspected by means of an agglomerative hierarchical clustering analysis based on the β_sim_ dissimilarity matrix, using command *hclust* (R Development Core Team 2013) with average linkage, in order to identify groups of territories with similar fauna. Thereafter, we explored the spatial and environmental correlates of the turnover pattern. A Constrained Analysis of Principal Coordinates (CAP) was computed in R using the vegan package (Oksanen et al. 2007) to examine the relationship between variation in the table of species occurrences and the three sets of predictor variables. CAP was selected because it can be computed with any dissimilarity index with ecological significance and, therefore, β_sim_ dissimilarity was preserved in the constrained ordination. Area, the nine aforementioned environmental variables, and spatial variables (the nine terms of a third degree polynomial of mean latitude and longitude, i.e. Trend Surface Analysis, see Legendre and Legendre 1998) were used as variables to perform constrained ordinations yielding respectively A, E and S models. Since the order of inclusion in the model affects the significance computed by the permutation tests (Borcard et al. 1992; Oksanen et al. 2013), the amount by which the explained variation was reduced due to the elimination of a single variable (compared with the complete model) was tested prior to the final analysis. This allowed the individual variables to be ranked in order of their independent contribution to the total variation in the response variable (from greatest to least), and the variables were included in the significance test in this order. Only significant variables were retained (p < 0.05) to avoid overfitting due to the inclusion of non-significant terms. Finally, variation partitioning among sets of variables was used to quantify the relative importance of the unique contributions of area (A), environment (E) and spatial variables (S), and their respective shared variances (Legendre and Legendre 1998).

*3. Variation in spatial turnover*. Given the observed patterns of spatial variation in species composition (i.e. spatial turnover), we assessed whether the rates of turnover with spatial distance were significantly different in northern and southern Europe. To do this, we transformed the pair-wise dissimilarity matrix based on β_sim_ on similarities (i.e. 1-β_sim_), and split the data into two groups: northern European countries, with mean latitude higher than 48 degrees (n=19), and southern European countries (n=17). Thereafter, we assessed the decay of assemblage similarity with spatial distance in Northern and Southern European datasets, using non-linear regression on similarity matrices to fit exponential decay curves expressed as *y*=a*e^-^*^bx^*, where *y* is similarity at distance *x*, *a* initial similarity and -*b* the rate of distance decay. Spatial distance was computed in km as the Euclidian distance between the UTM centroids of countries. Finally, to assess for significant differences in distance decay slopes between Northern and Southern regions, the frequency distributions of the parameters were estimated by bootstrapping. A frequency distribution of 1,000 slopes was retrieved by bootstrapping, using the boot package (Canty and Ripley 2008). When assessing the significance of one slope being larger in one region than in the other, the probability of obtaining the opposite result by chance was empirically computed by comparing the estimated distributions of parameters. The slopes found for Cryptocephalinae and Chrysomelinae were also compared to those of the European Cerambycidae, for which the diversity patterns were previously investigated (Baselga 2007; 2008; 2010).

## Results

### Variation in species richness

**Figure 1.**
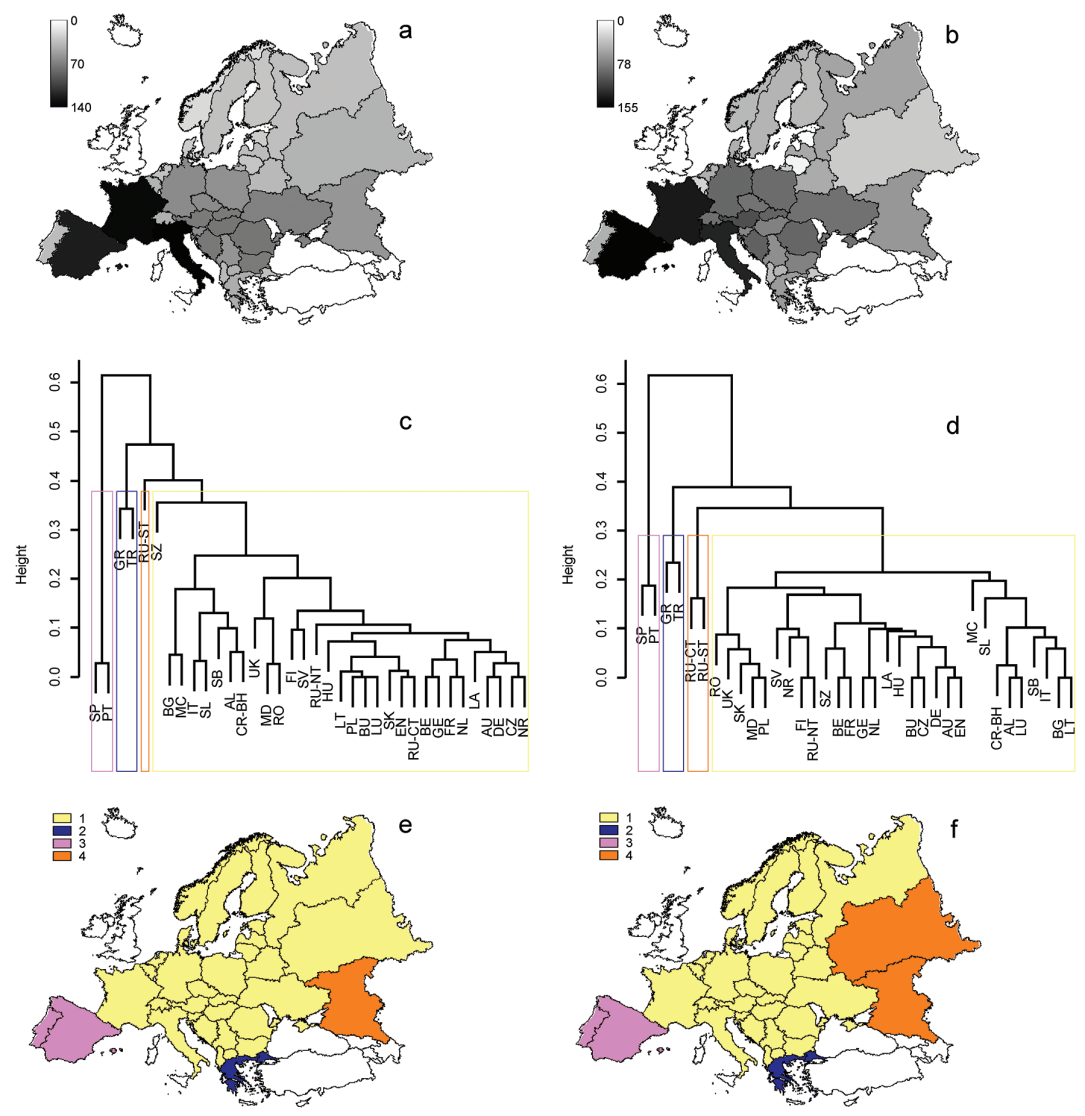
Patterns of variation in species richness (**a, b**), hierarchical clustering based in β_sim_ (**c, d**) and mapping of 4 major clusters (**e, f**) for cryptocephalines (left column: **a, c, e**) and chrysomelines (right column: **b, d, f**). Colours correspond to the 4 major clusters. Countries’ abbreviations follow those of Löbl and Smetana (2010).

**Figure 2.**
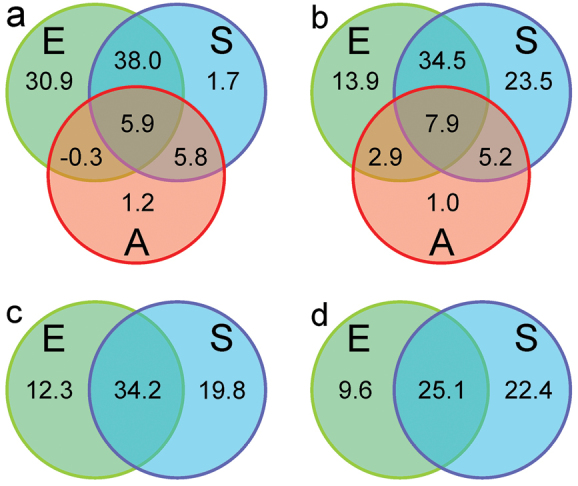
Partitioning of the variation (%) in species richness (**a, b**) and species composition (**c, d**) among groups of explanatory sets (A=area, E=environment and S=spatial variables) for European Cryptocephalinae (left column: **a, c**) and Chrysomelinae (right column: **b, d**).

The assessment of variation in species richness revealed a clear latitudinal gradient, with a significant reduction of diversity to the North in both groups (Figure 1a–b, Tables 1–2). In Cryptocephalinae, species richness was also significantly and positively related to country area, altitudinal range, annual mean temperature, maximum temperature of the warmest month, and spatial range of precipitation of driest quarter. In the case of mean altitude, longitudinal and latitudinal range and spatial range of annual precipitation the relationships were curvilinear, with maximum richness at intermediate values. After removing redundant variables, the spatial model for Cryptocephalinae comprised minimum latitude, and the quadratic function of longitudinal range, explaining 51.3% of the variance in species richness. The environmental model included maximum temperature of the warmest month, the spatial ranges of annual precipitation and precipitation of the driest quarter, and the quadratic function of altitudinal range, explaining 74.5% of the variance in richness. Variance partitioning showed that the largest portion of variation in richness (38.0%) was jointly explained by the environmental and spatial models, with a small contribution of country area (Figure 2a). The unique contribution of the environmental model (30.9%) was much larger than the unique contribution of the spatial model (1.7%).

**Table 1.** Relationships between cryptocephaline species richness and variables, and models for each group of variables. The sign of the relationships and percentage of explained variance (%) are shown, with their respective *F* parameters, degrees of freedom (d.f.) and p-values. A, S and E are the area, spatial and environmental models, respectively. *f^2^* is the second order polynomial of the variable considered.

Variable	Function	Variance (%)	F	d.f.	p
Area	logarithmic (+)	12.6	4.9	1, 34	0.034
Long	ns	6.0	2.2	1, 34	0.150
Long_min_	ns	10.0	3.8	1, 34	0.060
Long_max_	ns	4.0	1.4	1, 34	0.244
Long_ran_	quadratic (+,-)	21.9	4.6	2, 33	0.017
Lat	linear (-)	22.2	9.7	1, 34	0.004
Lat_min_	linear (-)	29.7	14.4	1, 34	0.001
Lat_max_	linear (-)	15.4	6.2	1, 34	0.018
Lat_ran_	quadratic (+,-)	16.7	3.3	2, 33	0.050
Alt	quadratic (+,-)	19.6	4.0	2, 33	0.027
Alt_ran_	linear (+)	28.3	13.4	1, 34	0.001
T_ann_	linear (+)	16.9	6.9	1, 34	0.013
T_ran_	linear (+)	30.1	14.6	1, 34	0.001
T_max_	linear (+)	16.5	6.7	1, 34	0.014
T_min_	ns	10.8	4.1	1, 34	0.050
P_ann_	ns	0.1	0.0	1, 34	0.894
P_ran_	quadratic (+,-)	31.8	7.7	2, 33	0.002
P_dri_	ns	0.7	0.3	1, 34	0.619
P_drn_	linear (+)	34.7	18.1	1, 34	<0.001
Model for A	log(Area)	12.6	4.9	1, 34	0.034
Model for E	T_max_ + P_ran_ + P_drn_ + *f^2^* Alt	76.1	19.2	5, 30	<0.001
Model for S	*f^2^* Long_ran_ + Lat_min_	51.3	11.3	3, 32	<0.001
Model S + A	*f^2^* Long_ran_ + Lat_min_ + log(Area)	52.3	8.5	4, 31	<0.001
Model E + S	T_max_ + P_ran_ + P_drn_ + *f^2^* Alt + *f^2^* Long_ran_ + Lat_min_	82.0	15.3	8, 27	<0.001
Model E + A	T_max_ + P_ran_ + P_drn_ + *f^2^* Alt + log(Area)	81.5	21.3	6, 29	<0.001
Model E + S +A	T_max_ + P_ran_ + P_drn_ + *f^2^* Alt + *f^2^* Long_ran_ + Lat_min_ + log(Area)	83.2	14.3	9, 26	<0.001

**Table 2.** Relationships between chrysomeline species richness and variables, and models for each group of variables. The sign of the relationships and percentage of explained variance (%) are shown, with their respective *F* parameters, degrees of freedom (d.f.) and p-values. A, S and E are the area, spatial and environmental models, respectively. *f^2^* is the second order polynomial of the variable considered.

Variable	Function	Variance (%)	F	d.f.	p
Area	logarithmic (+)	17.1	7.0	1, 34	0.012
Long	ns	9.9	3.7	1, 34	0.062
Long_min_	linear (-)	17.0	6.9	1, 34	0.013
Long_max_	ns	5.2	1.9	1, 34	0.179
Long_ran_	quadratic (+,-)	24.6	5.4	2, 33	0.009
Lat	linear (-)	14.3	5.7	1, 34	0.023
Lat_min_	linear (-)	19.4	8.2	1, 34	0.007
Lat_max_	ns	8.8	3.3	1, 34	0.079
Lat_ran_	quadratic (+,-)	23.3	5.0	2, 33	0.013
Alt	linear (+)	18.0	7.4	1, 34	0.010
Alt_ran_	quadratic (+,-)	41.1	11.5	2, 33	<0.001
T_ann_	ns	7.3	2.7	1, 34	0.110
T_ran_	linear (+)	33.9	17.4	1, 34	<0.001
T_max_	ns	5.0	1.8	1, 34	0.191
T_min_	ns	6.6	2.4	1, 34	0.129
P_ann_	ns	2.2	0.8	1, 34	0.385
P_ran_	quadratic (+,-)	43.0	12.4	2, 33	<0.001
P_dri_	ns	2.8	1.0	1, 34	0.331
P_drn_	linear (+)	44.5	27.3	1, 34	<0.001
Model for A	log(Area)	17.1	7.0	1, 34	0.012
Model for E	P_dri_ + *f^2^* P_ran_	60.0	16.0	3, 32	<0.001
Model for S	Long_min_ + *f^2^* Long_ran_ + Lat + Lat_ran_	71.2	14.8	5, 30	<0.001
Model S + A	Long_min_ + *f^2^* Long_ran_ + Lat + Lat_ran_ + log(Area)	75.1	14.6	6, 29	<0.001
Model E + S	P_dri_ + *f^2^* P_ran_ + Long_min_ + *f^2^* Long_ran_ + Lat + Lat_ran_	88.0	24.8	8, 27	<0.001
Model E + A	P_dri_ + *f^2^* P_ran_ + log(Area)	65.6	14.8	4, 31	<0.001
Model E + S +A	P_dri_ + *f^2^* P_ran_ + Long_min_ + *f^2^* Long_ran_ + Lat + Lat_ran_ + log(Area)	89.1	23.5	9, 26	<0.001

In Chrysomelinae, in addition to the negative relationship with latitude, species richness was also significantly and negatively related to minimum longitude and significantly and positively related to country area, mean altitude, annual mean temperature and spatial range of precipitation of driest quarter. In the case of longitudinal, latitudinal and altitudinal range and spatial range of annual precipitation the relationships were curvilinear, with maximum richness at intermediate values. After excluding collinear variables, the spatial model for Chrysomelinae consisted of mean latitude, latitudinal range, minimum longitude and the quadratic function of longitudinal range, explaining 71.2% of the variance in species richness. The environmental model comprised the spatial range of precipitation of the driest quarter, and the quadratic function of spatial range of annual precipitation, explaining 59.3% of the variance in richness. Variance partitioning showed that the largest portion of variation in richness (34.5%) was jointly explained by the environmental and spatial models, with a small contribution of country area (Figure 2b). The unique contribution of the spatial model (23.5%) was much larger than the unique contribution of the environmental model (13.9%).

### Variation in species composition

The assessment of variation in species composition (beta diversity) revealed the existence of similar patterns in both subfamilies, with the presence of singular faunas in the Iberian Peninsula, the Greek Peninsula, and Southern Russia, while the remaining European regions formed a relatively uniform cluster of countries with similar composition (Figure 1c–f). When the correlates of these patterns of variation in species composition were assessed, it turned out that faunal composition showed no significant relationship with species richness (pseudo-*F*_1,34_ = 1.10, p = 0.33 and pseudo-*F*_1,34_ = 0.66, p = 0.72, for Cryptocephalinae and Chrysomelinae, respectively). The same lack of significant relationship was found in both groups between species composition and the logarithm of country area (pseudo-*F*_1,34_ = 1.23, p = 0.29 and pseudo-*F*_1,34_ = 1.63, p = 0.11, respectively). In contrast, the assessment of the contribution of environmental and spatial variables to explaining beta diversity yielded significant contributions of both sets of variables (E and S models).

In Cryptocephalinae, the environmental model for variation in species composition included mean altitude, mean annual temperature, minimum temperature of the coldest month, and precipitation of the driest quarter as significant variables (pseudo-*F*_4,31_ = 6.73, p < 0.001) and explained 46.5% of the variation (Table 3). The spatial model consisted of the cubic polynomial of longitude, the quadratic polynomial of latitude and the interaction between longitude and latitude. To keep the number of variables balanced, only the first four terms with larger independent contribution (Lat, Long, Lat*Long and Long^2^) were included in the final spatial model (pseudo-*F*_4,31_ = 9.08, p < 0.001), which explained 54.0% of the variation. Partitioning of the variation in species composition among sets of variables showed that the largest fraction of variation (34.2%) was jointly explained by both models, and that the unique contribution of the spatial model (19.8%) was larger than the unique contribution of the environmental model (12.3%).

**Table 3.** Relationships between variation in cryptocephaline species composition (β_sim_) and variables, and models for each group of variables. Percentages of variation explained are shown, with their respective Pseudo-*F* parameters, degrees of freedom (d.f.) and p-values. S and E are the spatial and environmental models, respectively.

Variable	Variation (%)	Pseudo-*F*	d.f.	p
log(Area)	3.5	0.77	1, 34	0.288
Long	12.7	4.96	1, 34	0.002
Long^2^	6.3	2.30	1, 34	0.058
Long^3^	5.2	1.87	1, 34	0.094
Lat	25.1	11.37	1, 34	<0.001
Lat^2^	23.8	1.74	1, 34	0.085
Lat^3^	22.4	9.82	1, 34	<0.001
Long*Lat	11.4	4.37	1, 34	0.004
Long^2^*Lat	6.1	2.22	1, 34	0.067
Long*Lat^2^	11.4	4.35	1, 34	0.003
Alt	8.6	3.19	1, 34	0.023
Alt_ran_	3.6	1.28	1, 34	0.252
T_ann_	23.2	10.27	1, 34	<0.001
T_ran_	3.2	1.12	1, 34	0.339
T_max_	22.1	9.67	1, 34	<0.001
T_min_	18.6	7.79	1, 34	<0.001
P_ann_	6.1	2.22	1, 34	0.038
P_ran_	5.2	1.85	1, 34	0.089
P_dri_	6.0	2.17	1, 34	0.047
P_drn_	4.0	1.42	1, 34	0.196
Model for E	46.5	6.73	4, 31	<0.001
Model for S	54.0	9.08	4, 31	<0.001
Model E+S	66.3	6.64	8, 27	<0.001

In Chrysomelinae, the environmental model for variation in species composition included mean altitude, mean annual temperature, maximum temperature of the warmest month and minimum temperature of the coldest month (pseudo-*F*_4,31_ = 4.13, p < 0.001), explaining 34.7% of total variability (Table 4). The spatial model consisted of the quadratic function of longitude, latitude, the interaction between longitude and latitude and the interaction between longitude and the quadratic term of latitude. Only the first four ones with larger independent contribution (Long, Lat*Long, Long^2^ and Lat^2^*Long) were included in the final spatial model (pseudo-*F*_4,31_ = 7.01, p < 0.001), which explained 47.5% of the variation. Partitioning of the variation in species composition among sets of variables showed that the largest fraction of variation (25.1%) was again jointly explained by both models, but in Chrysomelinae the unique contribution of the spatial model (22.4%) was almost as large as the joint fraction, and more than two times the unique contribution of the environmental model (9.6%).

**Table 4.** Relationships between variation in chrysomeline species composition (β_sim_) and variables, and models for each group of variables. Percentages of variation explained are shown, with their respective Pseudo-*F* parameters, degrees of freedom (d.f.) and p-values. S and E are the spatial and environmental models, respectively.

Variable	Variation (%)	Pseudo-*F*	d.f.	p
log(Area)	5.2	1.88	1, 34	0.112
Long	13.6	5.36	1, 34	<0.001
Long^2^	8.2	3.03	1, 34	0.009
Long^3^	7.4	2.71	1, 34	0.042
Lat	17.6	7.25	1, 34	<0.001
Lat^2^	16.8	6.87	1, 34	<0.001
Lat^3^	16.0	6.48	1, 34	<0.001
Long*Lat	12.1	4.66	1, 34	<0.001
Long^2^*Lat	7.7	2.82	1, 34	0.027
Long*Lat^2^	10.9	4.18	1, 34	0.002
Alt	4.5	1.60	1, 34	0.119
Alt_ran_	3.5	1.24	1, 34	0.268
T_ann_	16.7	6.81	1, 34	<0.001
T_ran_	2.4	0.84	1, 34	0.564
T_max_	15.1	2.82	1, 34	<0.001
T_min_	15.2	6.08	1, 34	<0.001
P_ann_	4.9	1.76	1, 34	0.088
P_ran_	2.5	0.87	1, 34	0.525
P_dri_	4.9	1.76	1, 34	0.069
P_drn_	1.8	0.62	1, 34	0.794
Model for E	34.7	4.13	4, 31	<0.001
Model for S	47.5	7.01	4, 31	<0.001
Model E+S	57.1	4.49	8, 27	<0.001

### Variation in spatial turnover

**Figure 3.**
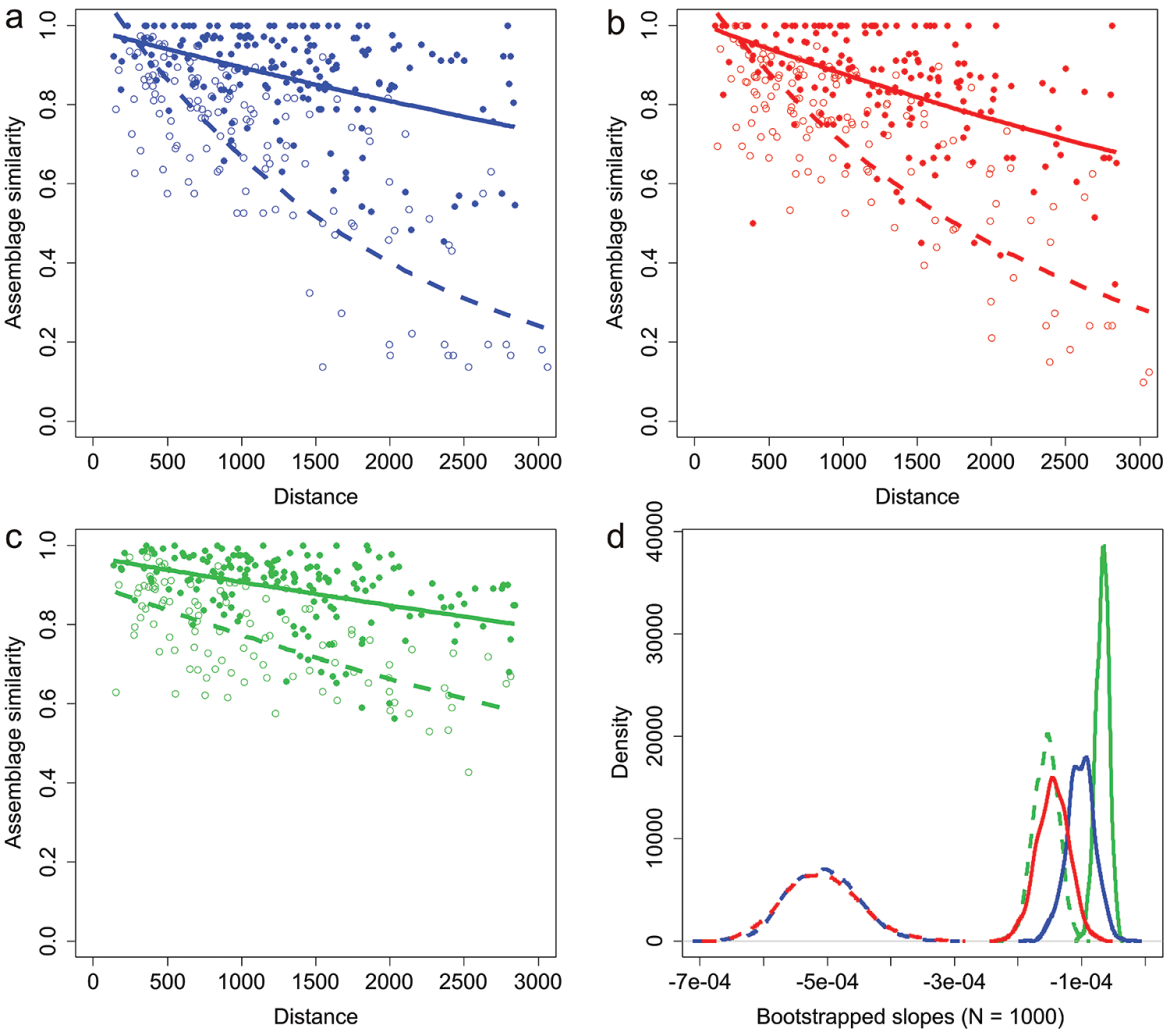
Distance decay of similarity with spatial distance in Northern Europe (solid dots, solid line) and southern Europe (hollow dots, dashed line) for cryptocephalines (**a**, blue), chrysomelines (**b**, red) and longhorn beetles (**c**, green). The density plots in (**d**) show the distribution of 1000 bootstrap replicates of the distance decay slopes (solid lines: northern Europe, dashed lines: southern Europe, colours corresponding to **a, b**, and **c**).

Assemblage similarity was significantly related to spatial distance in both subfamilies and European regions (Northern and Southern regions, Figure 3a–b), although the fit of exponential decay models was much better in the South (r^2^ = 0.57, p < 0.001 and r^2^ = 0.58, p < 0.001 for Cryptocephalinae and Chrysomelinae, respectively) than in the North (r^2^ = 0.16, p < 0.001 and r^2^ = 0.23, p < 0.001). Likewise, as shown in Figure 3d, the slopes of the distance-decay patterns in Southern Europe (*b* = -0.00051 and -0.00045 for Cryptocephalinae and Chrysomelinae, respectively) were significantly steeper (p < 0.001) than in Northern Europe (*b* = -0.00010 and -0.00014). Differences between distance-decay slopes in Cryptocephalinae and Chrysomelinae were neither significant in southern (p = 0.497) nor in northern Europe (p = 0.105). In contrast, the slopes of both Chrysomelinae and Cryptocephalinae in southern Europe were significantly steeper (p < 0.001) than that of the family Cerambycidae (Figure 3c–d).

## Discussion

The most prominent pattern in species richness of both cryptocephalines and chrysomelines is the existence of a clear latitudinal gradient. This is an almost universal macroecological pattern (Willig et al. 2003), that in Europe has been thoroughly documented in numerous taxa, especially vertebrates and plants (Araújo et al. 2008; Montoya et al. 2007; Rodríguez et al. 2005; Svenning and Skov 2007b; Whittaker et al. 2007) and some invertebrates such as butterflies (Hawkins and Porter 2003) and springtails (Ulrich and Fiera 2009), for example. Within Coleoptera, the situation is not very different and most taxa display a clear latitudinal gradient (Baselga 2008; Fattorini and Baselga 2012; Hortal et al. 2011; Schuldt and Assmann 2009). However, the steepness of the latitudinal richness gradient is subject of a great variation among different beetle taxa (Baselga et al. 2012b), with some taxa as families Scolytidae and Silphidae presenting weak (almost flat) gradients, while other taxa as genera *Trechus* (Carabidae) and *Otiorhynchus* (Curculionidae) having steep latitudinal gradients. This variation is partially explained by the differences in dispersal ability among beetle taxa, suggesting that limited post-glacial re-colonization processes are a major determinant of beetle richness gradients in Europe (Baselga et al. 2012b).

Other significant variables of species richness in both cryptocephalines and chrysomelines were country area, temperature, altitude and the range of precipitations. The major difference between both groups was the significant negative relationship between richness and minimum longitude in chrysomelines but not in cryptocephalines. Therefore, species richness models were qualitatively similar in both groups, although a relevant quantitative difference was also observed. While in cryptocephalines the environmental variables were relatively more important than the spatial ones (unique contributions of 31% vs. 2%, respectively), in chrysomelines the situation was reversed, with a largest contribution of spatial rather than environmental factors (unique contributions of 24% vs. 14%, respectively). These figures may be interpreted as cryptocephaline richness being more determined by environmental conditions (mostly temperature), while chrysomeline richness being more subject of undetermined factors causing a spatial structure independent of climatic factors, likely historical factors linked to diversification and limited dispersal. This interpretation is in accordance with the fact that flightless species are common among chrysomelines but not among cryptocephalines. In a wider context, the differences between species richness models for cryptocephalines and chrysomelines suggest that the preponderance of niche-based processes related to energy and water availability dynamics (Currie et al. 2004; Hawkins et al. 2003), niche conservatism and historical climate change (Hortal et al. 2011) or dispersal-based processes (Baselga et al. 2013; Svenning and Skov 2007a) is probably taxon-dependent.

The patterns of variation in species composition were, in contrast, very similar in both groups: several European southern regions, namely Iberian and Greek peninsulas and southern Russia, presented singular species composition differing from the rest of the continent, which harboured a relatively homogeneous fauna. Therefore, the environmental correlates of variation in species composition were very similar in both groups, including altitude and several temperature-related variables. The only difference was the significant relationship between species composition and precipitation of the driest quarter in cryptocephalines but not in chrysomelines, suggesting that cryptocephaline species are filtered by water availability but chrysomeline species are not. Likewise, the spatial determinants are similar in both groups, including both latitudinal and longitudinal components. The partition of variation in species composition between environmental and spatial variables revealed that a strong spatial structure (independent of environmental factors) exist in both groups, as also found in other beetle taxa (Baselga 2008; Fattorini and Baselga 2012). This suggests that, besides the effect of environmental factors causing species turnover as a result of differential physiological requirements, other spatially-structured processes have deeply influenced the replacement of cryptocephaline and chrysomeline faunas across Europe. Speciation processes and limited dispersal are likely behind such spatial patterns, and the effect of Pleistocene glaciations appears as one of the most plausible explanations. Indeed, while northern European faunas were obliterated during Pleistocene glaciations, southern European regions have acted as faunal refugia during glacial cycles. This has impacted southern European faunas in two ways. In first place, pre-Pleistocene assemblages were not obliterated, providing more time for in-situ diversification (Hewitt 2004), compared to northern regions where beetle assemblages are necessarily recent (i.e. arrived there since last glaciation). Indeed, several studies have reported the pre-Pleistocene origin of Mediterranean endemics (Andújar et al. 2012; Condamine et al. 2013; Hidalgo-Galiana and Ribera 2011; Ribera et al. 2010). In second place, glacial refugia have also acted as speciation centres (Gomez-Zurita et al. 2012; Ribera and Vogler 2004), increasing the singularity of southern faunas. Among the different glacial refugia that could serve as sources of re-colonization of northern regions, the Italian peninsula is the only one that does not present a markedly singular fauna (despite its high species richness), a fact also observed on longhorn and darkling beetles (Baselga 2008; Fattorini and Baselga 2012). This might suggest that the Italian peninsula was one of the major re-colonization sources, making most of the European faunas similar to the Italian one, but less so to the Iberian and Greek assemblages, in which re-colonization events seem to have been much more limited. Finally, the higher relevance of spatial factors for species composition compared to species richness suggests that species composition is more linked to dispersal and historical contingencies than species richness, which would be more controlled by environmental limitations.

The effects of Pleistocene glaciations seem also to be behind the differences in the patterns of distance decay of similarity between northern and southern regions. As it could be predicted from the visual inspection of maps illustrating the distribution of major clusters, the spatial turnover of cryptocephaline and chrysomeline assemblages with spatial distance is much steeper in southern Europe compared to northern Europe. As previously suggested elsewhere (Baselga 2010; Baselga et al. 2012a), this difference could be interpreted as a consequence of Pleistocene glaciations. Southern regions have retained beetle assemblages during larger periods of time, allowing speciation processes to accumulate and thus increasing the differences between assemblages. In contrast, beetle assemblages in northern regions were obliterated during glaciations, and the species currently present there are all generalist ones with high dispersal ability (which have allowed them to colonize the continent from southern refugia). Interestingly, differences between northern and southern slopes of the distance decay pattern are much larger in both leaf beetle taxa than in longhorn beetles. The reason for this larger difference is that, in southern Europe, leaf beetles present a much steeper distance decay of similarity than longhorn beetles. In contrast, the decay of similarity with spatial distance in northern Europe is more similar for the three taxa. In other words, differences in diversity patterns between the two leaf beetle taxa and the longhorn beetles are more marked in southern than in northern Europe, suggesting again that the relative homogeneity of northern faunas is a phenomenon resulting from a common process in all taxa, likely post-glacial re-colonization by only those species with high dispersal ability, in line with previous evidence (Baselga et al. 2012b). In contrast, southern faunas would be composed by older assemblages and differences in diversity patterns of taxa had more time to accumulate, reflecting dispersal, ecological and evolutionary differences.
